# Resveratrol Supported on Magnesium DiHydroxide (Resv@MDH) Represents an Oral Formulation of Resveratrol With Better Gastric Absorption and Bioavailability Respect to Pure Resveratrol

**DOI:** 10.3389/fnut.2020.570047

**Published:** 2020-11-13

**Authors:** Rossana Giulietta Iannitti, Alessandro Floridi, Andrea Lazzarini, Alice Tantucci, Roberta Russo, Francesco Ragonese, Lorenzo Monarca, Concetta Caglioti, Roberto Spogli, Lucio Leonardi, Massimiliano De Angelis, Federico Palazzetti, Bernard Fioretti

**Affiliations:** ^1^S&R Farmaceutici S.p.A., Perugia, Italy; ^2^Forensic Toxicology Laboratory, CRABioN Research Center, Perugia, Italy; ^3^Department of Medicine, University of Perugia, Perugia, Italy; ^4^Department of Chemistry, Biology, and Biotechnologies, University of Perugia, Perugia, Italy; ^5^Prolabin & Tefarm, Spin-Off Un. of University of Perugia, Perugia, Italy

**Keywords:** resveratrol, human health, bioavailability, dissolution rate, whole blood concentration

## Abstract

Resveratrol attracts great interest because of the plethora of *in vitro* effects at the micromolar concentration range. Unfortunately, these effects are difficult to establish *in vivo*, due to the low concentration of resveratrol reached. This discrepancy is due to the molecules low solubility in water that favors the propensity for an intestinal absorption rather than in the gastric compartment. To address these challenges, we developed a Solid Dispersion of Resveratrol Supported by Magnesium Di Hydroxide formulation (Resv@MDH). This formulation displays increased water solubility and better bioavailability relative to pure resveratrol in the rabbit animal model. In our study, we evaluated the pharmacokinetics profile of resveratrol in six healthy human subjects following 180 mg of oral resveratrol administration, derived from Resv@MDH or pure resveratrol. Free resveratrol was evaluated in the whole blood sample by using HPLC - MS/MS. In comparison with pure resveratrol that displays an increase of the maximum plasma concentration, Cmax at about 90 min and 2 μM, Resv@MDH displays an earlier peak of resveratrol concentration with an increase of Cmax at about 30 min and 6 μM. The different kinetics suggest a main gastric route for resveratrol absorption from Resv@MDH, where, because of its improved dissolution rate, there seems to be a higher propensity for an acidic environment, as opposed to that with pure resveratrol. This conclusion is also supported by the numerical simulation analysis, which considers the principal steps during the oral route administration. Moreover, there is a 2-fold increase in the amount of free resveratrol with respect to pure resveratrol confirming a better bioavailability observed in the animal model. The characteristic feature of the pharmacokinetic profile of Resv@MDH implies that the beneficial properties of resveratrol in human health should be capitalized on it.

## Introduction

Resveratrol [(E)-5-(p-hydroxystyryl) resorcinol] is a polyphenol with a stilbenic structure and is widely diffused in nature as phytoalexin (1). Biological properties of resveratrol have mostly been related to its antioxidant effects, due to its polyphenolic nature, and mainly to its ability to increase the expression of the intracellular antioxidant mechanism (e.g., SOD, CAT, and GSH). Along with this protective effect on oxidative stress, resveratrol has been shown to promote the mitochondrial metabolism by increasing the number of mitochondria (mitochondrial biogenesis), their activity and production of ATP. Resveratrol exerts its biological effects thought AMPK/SIRT1/Nrf2, ERK/p38 MAPK, and PTEN/Akt- signaling pathways (1–3). Resveratrol has also been shown to have several beneficial properties for human health such as protection from metabolic and cardiovascular diseases, neuroprotection, and both anti-inflammatory and immunomodulation effects (1, 4). Efficacy associated with resveratrol use has been attributed to the activation of the lysine deacetylase, Sirtiun 1 (SIRT1) (5), the cAMP pathway, or the AMP-activated protein kinase (AMPK) (6, 7) and partial agonist in estrogen receptors (8). For this reason, resveratrol has a potential application in the prevention and treatment of chronic diseases, including cancer (9), neurodegenerative diseases such as Alzheimer's (9), and metabolic diseases such as diabetes (10), as well as anti-aging effects. The therapeutic properties of resveratrol are promptly exploited by the pharmaceutical industry and different conventional oral dosage forms have been developed (11).

The efficacy, safety, and pharmacokinetics of resveratrol have been documented in hundreds of clinical trials, with additional trials currently ongoing. A note however is that some of these clinical trials have not yet been published (12). Nevertheless, published trials represent a relatively small portion when one considers the thousands of published reports to date that prove the many *in vitro* effects of resveratrol and its rapid metabolism in the human body, which can limit its clinical use and effectiveness. The bioavailability of oral administration of resveratrol is limited, mainly due to its physical and chemical properties (water solubility) and its biological stability (metabolism). Given resveratrol's low solubility in water and high membrane permeability, it is collocated in the second class of the biopharmaceutical classification system [BCS, (13, 14)]. Due to its chemical and physical profile, after oral administration, resveratrol is slowly absorbed along the gut. The principal site of absorption is at the intestinal level through passive diffusion or active transport via ATP-dependent binding cassette (ABC) transporters (15). Resveratrol can be absorbed through the portal bloodstream to the liver by passive diffusion or receptor-mediated transport, where it is rapidly metabolized in glucuronide and sulfate derivatives. Additionally, resveratrol can efficiently bind in a non-covalent manner, to proteins such as albumin, lipoproteins and, in particular, to a fraction of low-density lipoproteins (LDLs) (16). As a result, plasma-free resveratrol is quite limited due to its short plasma half-life (t1/2) (17) and extensive metabolism in the intestine and liver compartments.

The pharmacokinetics of resveratrol has been investigated in several clinical studies; with various oral doses and regime of administration such as single dose or regime protocols (11). Among single dose studies, Almeida et al. (18) evaluated the pharmacokinetics profile after 25, 50, 100, and 150 mg of resveratrol administration whereas Nunes et al. (19), Vaz-da-Silva et al. (20) and Sergides et al. (15) study the administration of a dose of 200, 400, and 500 mg of resveratrol, respectively. Altogether, the principal results from these studies are that the resveratrol plasma concentration reached *in vivo* is in the submicromolar range with a time peak concentration of about 1 h according to a principal intestinal site of absorption (11). Recently, by using an animal model, a higher distribution of resveratrol in cellular compartment (76%) of blood respect to that in plasma was observed (21). This aspect could be taken into account to estimate the pharmacokinetic properties of single dose of oral resveratrol (15).

Several strategies have been designed to modify resveratrol's pharmacokinetics, thereby increasing its bioavailability and improving its potential health benefits. Recently, we have proposed a solid dispersion of resveratrol on magnesium dihydroxide, Resv@MDH. This strategy increases its solubility and bioavailability, demonstrating its potential use in enhancing the biological properties of resveratrol *in vivo*. Compared with pure resveratrol, Resv@MDH solubilizes itself faster and in greater amounts, has an increased bioavailability demonstrated in the rabbit animal model and thus proving itself advantageous in biopharmaceutical terms (22). The purpose of this study is to assess the pharmacokinetic profile of resveratrol in healthy human subjects by estimating resveratrol from whole blood and comparing it to pure resveratrol from Resv@MDH.

## Materials and Methods

### Materials

Acetonitrile, Ultrapure water, Formic acid and resveratrol used are of analytical grade for LC/MS and purchased from the company SIGMA (Milan, Italy) and/or Carlo Erba (Milan, Italy). The resveratrol used for the Resv@MDH preparation and pure resveratrol were obtained from the Polygonum cuspidatum Siebold & Zucc extract (98% pure). Resv@MDH was obtained by Good Manufacturing Practice (GMP) chain (Prolabin & Tefarm, Perugia, Italy and La Sorgente del Benessere S.p.A, Fiuggi Italy). Resv@MDH is distributed by S&R Farmaceutici S.p.A. Bastia Umbra, Italy, with the trade name of Revifast®. The resveratrol content in Resv@MDH was evaluated using the HPLC method and the mean value obtained in the three samples was about 30 and 70% of total weight of resveratrol and magnesium dihydroxide, respectively.

### Dissolution Assays in Sink Condition

Dissolution assay was performed in sink condition to better reproduce the *in vivo* situation. The general procedure used was previously described (22). Briefly, the weighed amount of Resv@MDH or resveratrol was placed in series of closed flat-bottomed glass vessels containing 250 mL of Simulated Gastric Fluid (SGF). The composition of SGF was 35 mM of NaCl, pH 1.2 with HCl. The vessels were inserted in shaking water bath (Nuve ST 30) at 37°C and 110 rpm for 2 h. At appropriate times, the reaction was sampled, filtered (Spartan 13/02 RC, Whatman GmbH, Dassel, Germany) and analyzed by HPLC. The measurements were performed by an Agilent HPLC 1200 series equipped with an Agilent Zorbax SB C18 4.6 × 250 mm 5-μm Agilent P/N 880975-902 column and VWD Detector, λ = 306 nm. Elution was carried out under isocratic conditions using as mobile phase (Water + 0.1% v/v Trifluoroacetic acid)/(Acetonitrile + 0.1% v/v Trifluoroacetic acid) = 65/35, with a flow of 1 mL/min and a column temperature of 30°C. For the quantification of resveratrol, a calibration was performed to detect the polyphenol at a retention time of 5.6 min with a detection limit of 4 ng/mL.

### Study Design and Treatments

Six healthy adults aged 18–41 years of both sexes with body mass index > 18.5 and <32 kg m^−2^ were eligible for the study if they were willing and able to understand and sign an informed consent (Table 1). Inclusion criteria: non-smokers, not on any pharmacological therapy and overall in good health as determined by medical history review, physical examination, and clinical laboratory tests. Key exclusion criteria included pregnancy or lactation, history of hypersensitivity reactions, under drug treatment or dependency or alcohol abuse. Subjects taking dietary supplements containing antioxidants, untreated hypothyroidism were also excluded.

**Table 1:** Demographic characteristics of the study population.

**Patient no**	**Age**	**BMI**	**Systolic BP, mmHg**	**Diastolic BP, mmHg**
1	32	21.4	105	65
2	41	32	121	82
3	32	26	119	74
4	40	26	123	73
5	31	28	117.5	75
6	35	20.4	107	70

**Figure 1:**
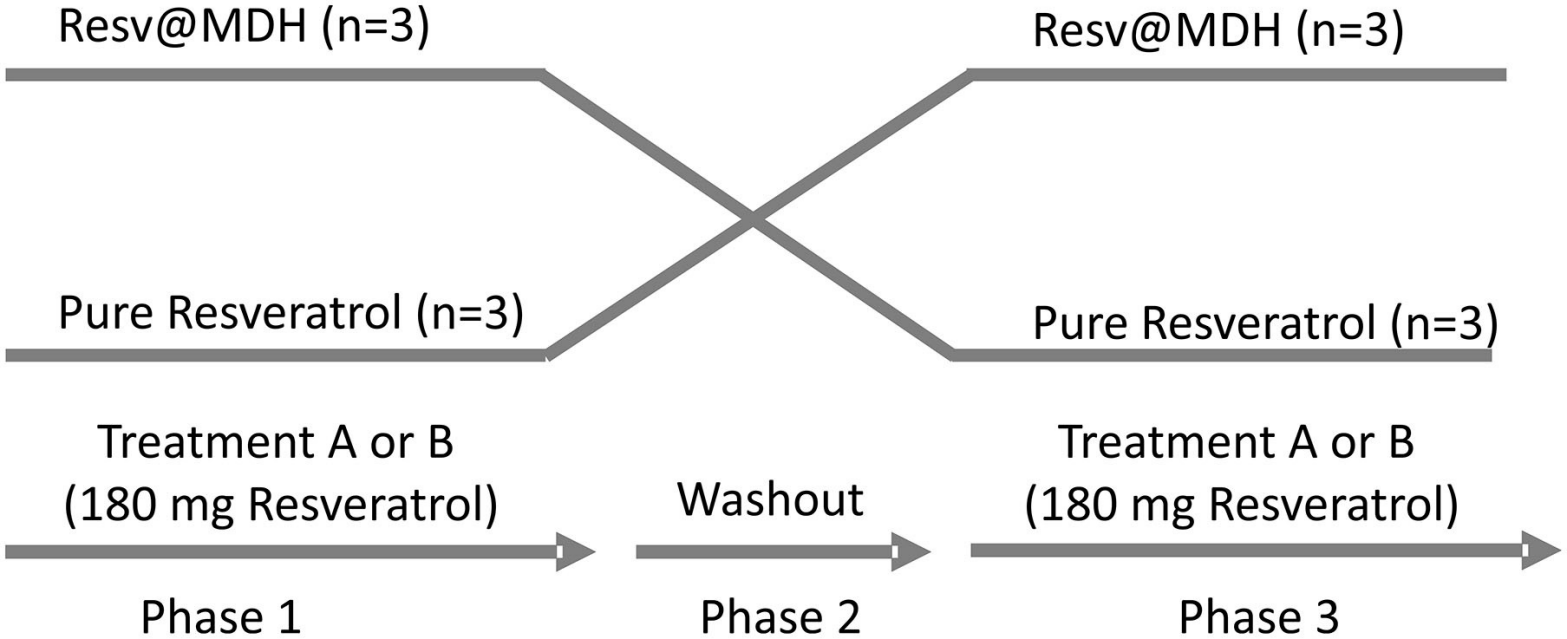
Study design. Randomized, open label, single blind crossover study. Two single-dose treatments of resveratrol were administered orally with a 1-day intermediate washout period. The treatments consist of a solution of either (A) 180 mg pure Resveratrol (Polygonum cuspidatum 98%) or (B) 180 mg Resveratrol from Resv@MDH (600 mg 30% w/w of resveratrol) dispersed in water. After a 1-day washout, subjects who took solution A took solution B and vice versa. No drop out of patients was recording during the trial.

The study was a randomized, single blind, crossover study carried out in a single center in Perugia, Italy. Two single-dose treatments were administered orally under fasting conditions. Phase 1: subjects were given on the first day, a solution of content A or B (see above) obtained by dissolving the contents of the capsule in water. Samples of 2 ml of venous blood were taken in a tube containing EDTA, from the arm vein via ago cannula, at the following times 0 min (fasting before administration), 15, 30, 60, 90, 120, and 180 min. Phase 2: second day, wash-out period: each treatment period was separated by a 1-day washout. Phase 3: during the third day, the person who took solution A in the first day, takes solution B, and *vice versa*. Blood samples were taken as described for Phase 1. See Study design (Figure 1).

The treatment sequence for each participant was assigned by a computer-generated randomization list. The treatments were in the form of a suspension obtained by adding the content of capsule containing 180 mg of resveratrol in either the Resv@MDH form or pure resveratrol to water. Subjects fasted overnight for at least 10 h prior to the suspension of resveratrol tablet administration. The study protocol was approved by a regional ethics committee review board (project number 12477/18, title approved on 18/01/2018 by CEAS Umbria, Perugia Italy) and registered in the ClinicalTrials.gov Protocol Registration System (identifier: NCT04258306). The study was conducted in accordance with Good Clinical Practice, and followed the requirements of the Declaration of Helsinki and relevant European regulation and directives. All subjects provided written informed consent.

### Pharmacokinetic Profile Quantification

Pharmacokinetic profile quantification was performed using HPLC-MS/MS applied to the whole blood sample to evaluate resveratrol (21, 23). Specifically, the blood samples were diluted with acetonitrile in a ratio of 1: 4, vortexed and centrifuged at 14,500 rpm for 5 min. The supernatant was filtered using a 0.2 μm membrane filter before injecting in HPLC coupled to triple quadrupole linear MS/MS (AB sciex 5500, Shimadzu). Separation was achieved on a C18 column (Agilent Eclipse Plus C18 3.5 μm 4.6 × 100 mm PN 959961-902, SN USUXR18880, LN B14112) at 35°C with a flow rate of 0.9 ml/min. The gradient elution system (A: water and 0.1%; formic acid B: acetonitrile and 0.1%; formic acid) was as follows: 0–2 min (2% B and 98% A), 2–7 min (100% B), 7–10 min (2% B and 98% A). Retention time and the Selected Reaction Monotoring (SRM) transition were developed by using analytical grade resveratrol (Sigma-Aldrich, Milan). In the sample, Resveratrol was quantified by monitoring SRM transition at m/z 229 → 165. The validation of the analytic methods was performed by adding a known amount of resveratrol in the blood samples. The Lower limit of quantitation (LLOQ) was 5 ng/ml.

### Statistical Analysis

All results are expressed as the mean ± SE. Differences between two related parameters were assessed by Student's *t*-test. Differences at ^*^p < 0.05 were considered significant.

## Results

### The Dissolution Rates of Resv@MDH and Pure Resveratrol

**Figure 2:**
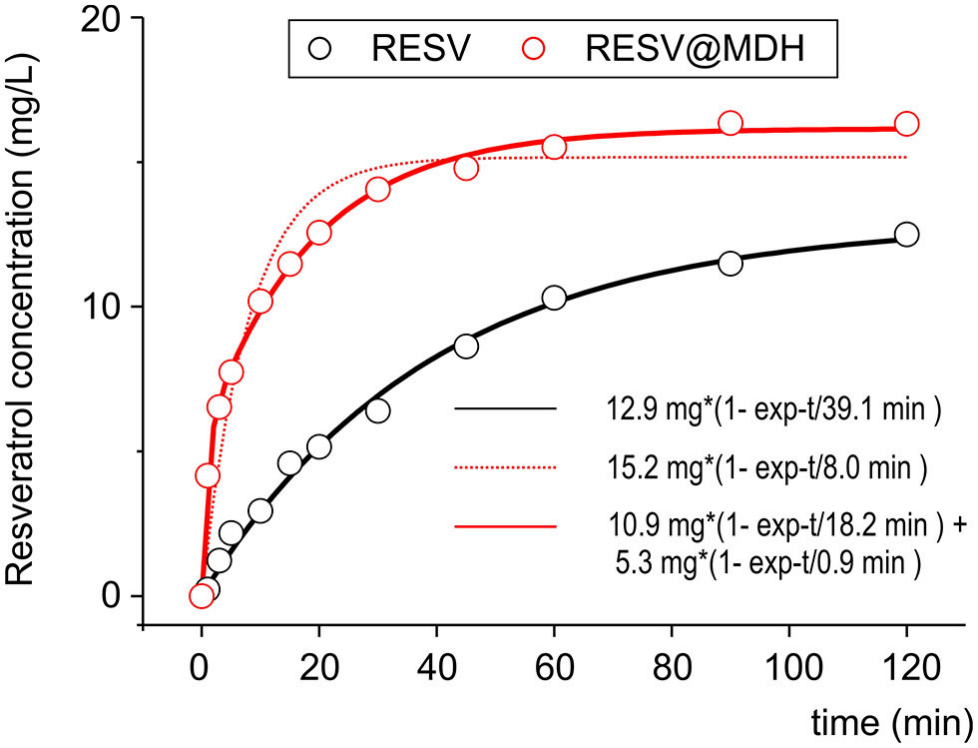
Dissolution profile of resveratrol and Resv@MDH in gastric simulation fluid. The plot reports the solute concentration in mg/L vs. time in minutes. Red dots and black dots indicate the experimental data of Resv@MDH and resveratrol, respectively. The solid red line, black line and red dash line represent the best fits with monoexponential models (black and red dots) and biexponential (red line), respectively, as described in the text.

In our previous work, we compared the dissolution rate of resveratrol from Resv@MDH with that of pure resveratrol in a condition that predicted the saturation conditions (22). To better investigate the pharmacokinetics of resveratrol following oral administration, we then studied the dissolution rate in sink conditions [(24), no oversaturation condition]. Figure 2 shows the dissolution profiles of 20 mg of resveratrol in a liter of solution (pure resveratrol vs. Resv@MDH) such an amount is three times lower than the solubility of resveratrol in the used dissolution liquid (max solubility 62 mg/L). The experimental data relevant to the dissolution of pure resveratrol is best described through mono-exponential kinetics, with constant dissolution of about 39 min. In contrast, mono-exponential kinetics (dashed red lines) are required to describe the dissolution data of resveratrol from Resv@MDH. In this case, bi-exponential kinetics are required (*R*^2^ of 0.94 and 0.99 for mono and bi-exponential models, respectively), where there is a fast component of about 1 min (about 5 mg) and a slower one of about 18 min (about 10 mg). The ratio of the fast component over the slower one is 1:2. These dissolution profiles are consistent with the micro-particle composition of Resv@MDH (22).

The dissolution process of the pure resveratrol is given by

(1) $$\begin{array}{r} {\text{R}}_{\text{c}}⇄{\text{R}}_{\text{sol}} \end{array}$$

where R_c_ is the solid crystal resveratrol and R_sol_ is the soluble form (hydrated). It follows a first-order kinetic, given by the equation

(2) $$\begin{array}{r} \frac{\text{d}\left[{\text{R}}_{\text{sol}}\right]}{\text{dt}}={\text{k}}_{\text{d}1}*\left(\left[{\text{R}}_{\max}\right]-\left[{\text{R}}_{\text{sol}}\right]\right) \end{array}$$

where [R_sol_] is the concentration in water, *t* is the time, *k*_*d*1_ = 2.56·10^−2^ min^−1^ is the kinetic constant dissolution 1 and [R_max_] = 12.9 mg/L is the asymptotic concentration of resveratrol in water obtained by fit.

Concerning the dissolution of Resv@MDH in water, we found that it follows two kinetic laws, arguably given by two different dissolution processes, according to the following scheme: 
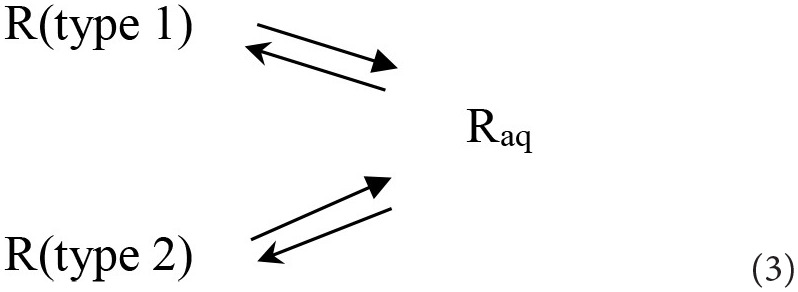


where R (type 1) and R (type 2) are two different forms of resveratrol, namely, R (type 1) is crystalline pure resveratrol, while R (type 2) is formed by resveratrol and brucite agglomeration according to a previous report (22). Both dissolution processes follow a first-order kinetic, given by the following equations:

(4) $$\begin{array}{r} \frac{\text{d}\left[{\text{R}}_{\text{sol}}\right]}{\text{dt}}={\text{k}}_{\text{dtype}1}*\left(\left[{\text{Rtype}1}_{\max}\right]-\left[{\text{R}}_{\text{sol}}\right]\right) \end{array}$$

(5) $$\begin{array}{r} \frac{\text{d}\left[{\text{R}}_{\text{aqsol}}\right]}{\text{dt}}={\text{k}}_{\text{dtype}2}*\left(\left[{\text{Rtype}2}_{\max}\right]-\left[{\text{R}}_{\text{sol}}\right]\right) \end{array}$$

where *k*_*dtype*1_ = 5.48·10^−2^ min^−1^ and k_dtype2_ = 1.10 min^−1^ are, respectively the kinetic constant of dissolution of R (type 1) and R (type 2), while [Rtype1_max_] = 10.9 mg/L and [Rtype2_max_] = 5.3 mg/L are the asymptotic concentration in water of the two forms of resveratrol. The dissolution curve of Resv@MDH in Figure 2 is given by the sum of Equations (4) and (5).

The dissolution processes have been indicated as an equilibrium processes; thus, the dissolution kinetics must be intended as a resultant of the direct and -reverse processes. This aspect however, must be investigated in future studies.

### Pharmacokinetic Properties of Resv@MDH and Pure Resveratrol After Oral Administration

**Figure 3:**
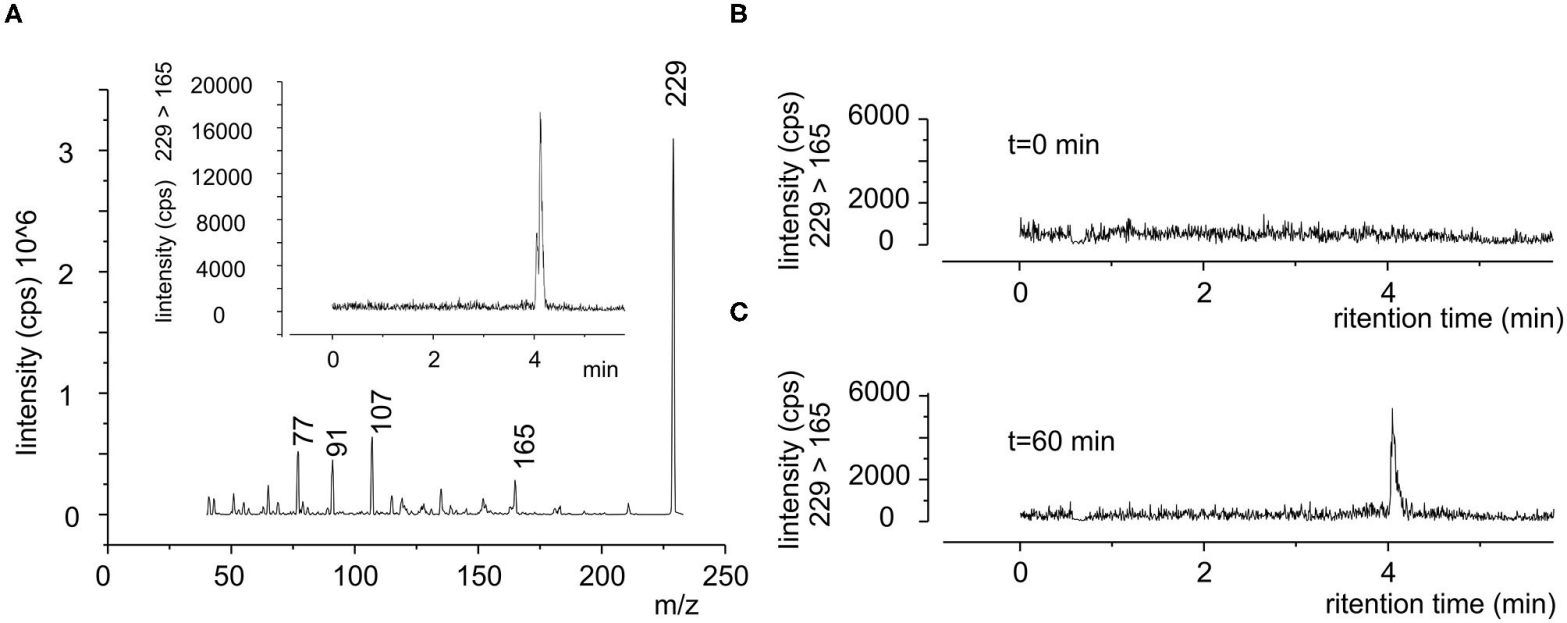
Typical SRM chromatogram of resveratrol in human blood. **(A)** Product ion mass spectra of standard resveratrol (100 ng/ml), where it is possible to see the principal m/z ratio of 229, 165, 107, 91, and 77 peaks. Inset chromatograms of 229-165 SRM in the same sample, shown is the retention time of resveratrol. **(B,C)** Blood sample obtained from same subject (subject 1) before (0 min) and after 60 min after the oral administration of a single dose of pure resveratrol (180 mg), respectively.

Since resveratrol cannot be completely recovered from plasma (21), we directly evaluated the polyphenol in the whole blood by using LC/MS-MS. Figure 3A, displays the mass spectrum of standard resveratrol solution (100 ng/ml), where the principal fragmentation pattern is apparent. We selected SRM (229-165) to evaluate the retention properties of resveratrol and defined it at about 4 min (inset Figure 3A), though a similar result was obtained following other SRM (data not shown). As expected, in the blood sample of fasting subjects no resveratrol trace was observed (Figure 3B), whereas it was clearly evident after oral administration of pure resveratrol (Figure 3C). The resveratrol concentration in the plasma was obtained from the calibration curve and expressed in μM units (see Methods).

**Figure 4:**
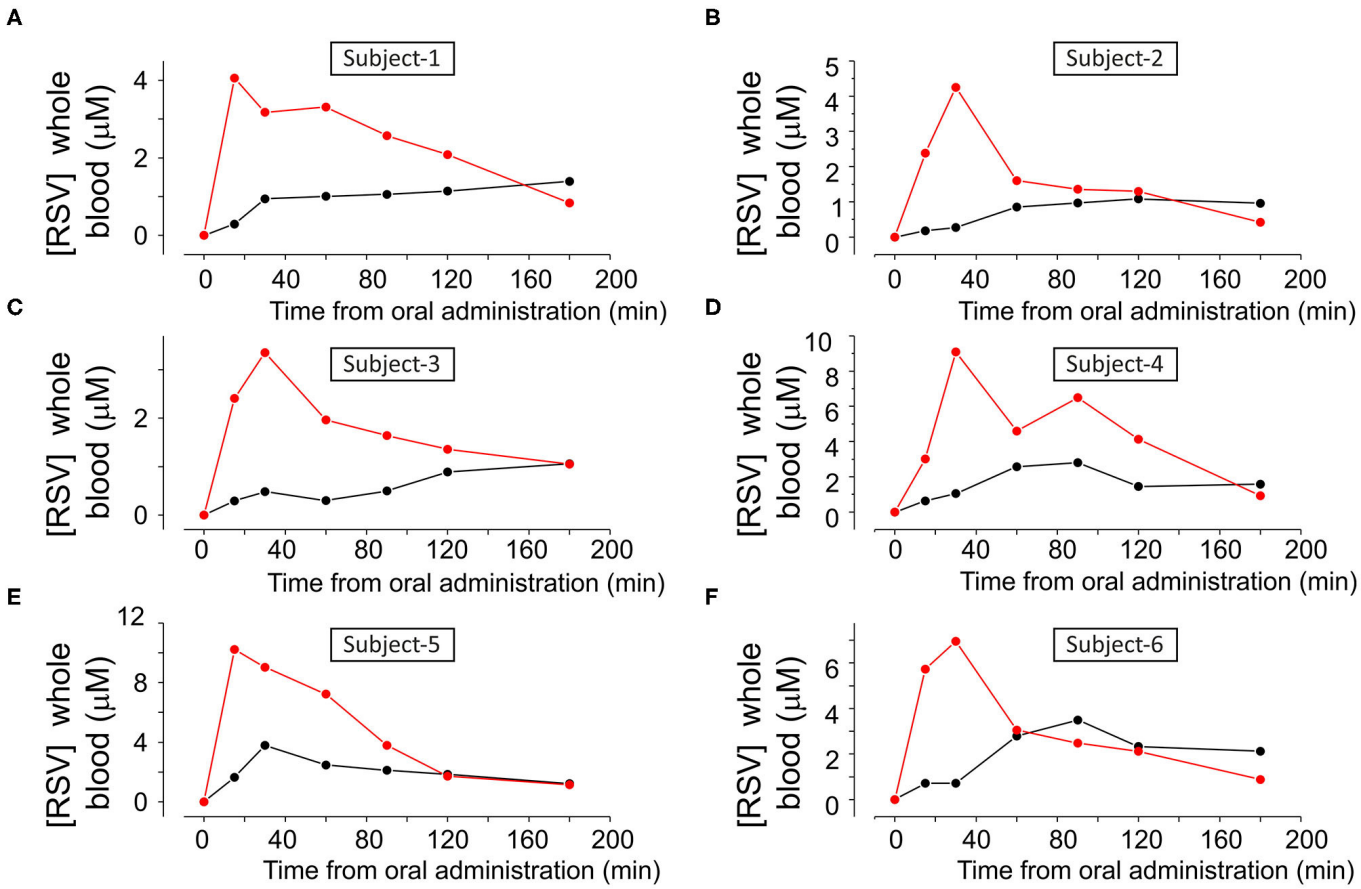
**(A–F)** Concentration of resveratrol in whole blood as a function of the time, in each subject after treatment with either **(A)** 180 mg pure Resveratrol (Polygonum cuspidatum 98%, (black dots) or **(B)** 180 mg Resveratrol from Resv@MDH after oral administration (red dots).

Figures 4A–F and Supplementary Figure 1 show the single data points of resveratrol in blood samples obtained from the six subjects that participated in the study following oral administration of 180 mg of resveratrol from pure resveratrol (black dots) and Resv@MDH (red dots), respectively. Resveratrol concentration was defined based on a calibration curve by using chromatographic methods described in Figure 3. Before the oral administration of either form of resveratrol (*t* = 0), the blood concentration of resveratrol was undetectable indicating also a sufficient wash out period. In all subjects, the blood concentration of resveratrol following Resv@MDH administration was higher than that with pure resveratrol. This observation held true up until the 2-h mark after oral administration, at which point differences between blood concentration of Resv@MDH and pure resveratrol ceased to exist. By using PK solve software (25), we analyzed the pharmacokinetic properties of the plasma profile from each subject. Parameters derived from this analysis are summarized in Table 2.

**Table 2:** Summary of pharmacokinetic parameters for resveratrol following a single dose of 180 mg resveratrol.

**Parameters**	**Unit**	**Mean resveratrol**	**se**	***n***	**Mean Resv@MDH**	**se**	***n***	***P***
t1/2	Min	128.9	6.5	3	63.6	14.6	6	*p* = 0.02
Tmax	Min	115	23.8	6	25	3.2	6	*p* = 0.004
Cmax	μmol/ml	2.3	0.5	6	6.3	1.2	6	*p* = 0.010
AUC 0–3	νmol/ml*min	248.3	48.7	6	526.0	92.8	6	*p* = 0.024

*The parameters are calculated as follows: AUC 0–3, which is the area under the curve that was integrated from the plasma concentrations between time 0 h and the 3 h after oral administration of the resveratrol; Cmax, is the maximum plasma concentration, obtained directly from the data without interpolation; Tmax, is the time to reach the Cmax, obtained directly from the data without interpolation; T1/2, is the plasma half life. All parameter, definitions, and statistical aspects are in accordance to Zhang et al. (25)*.

**Figure 5:**
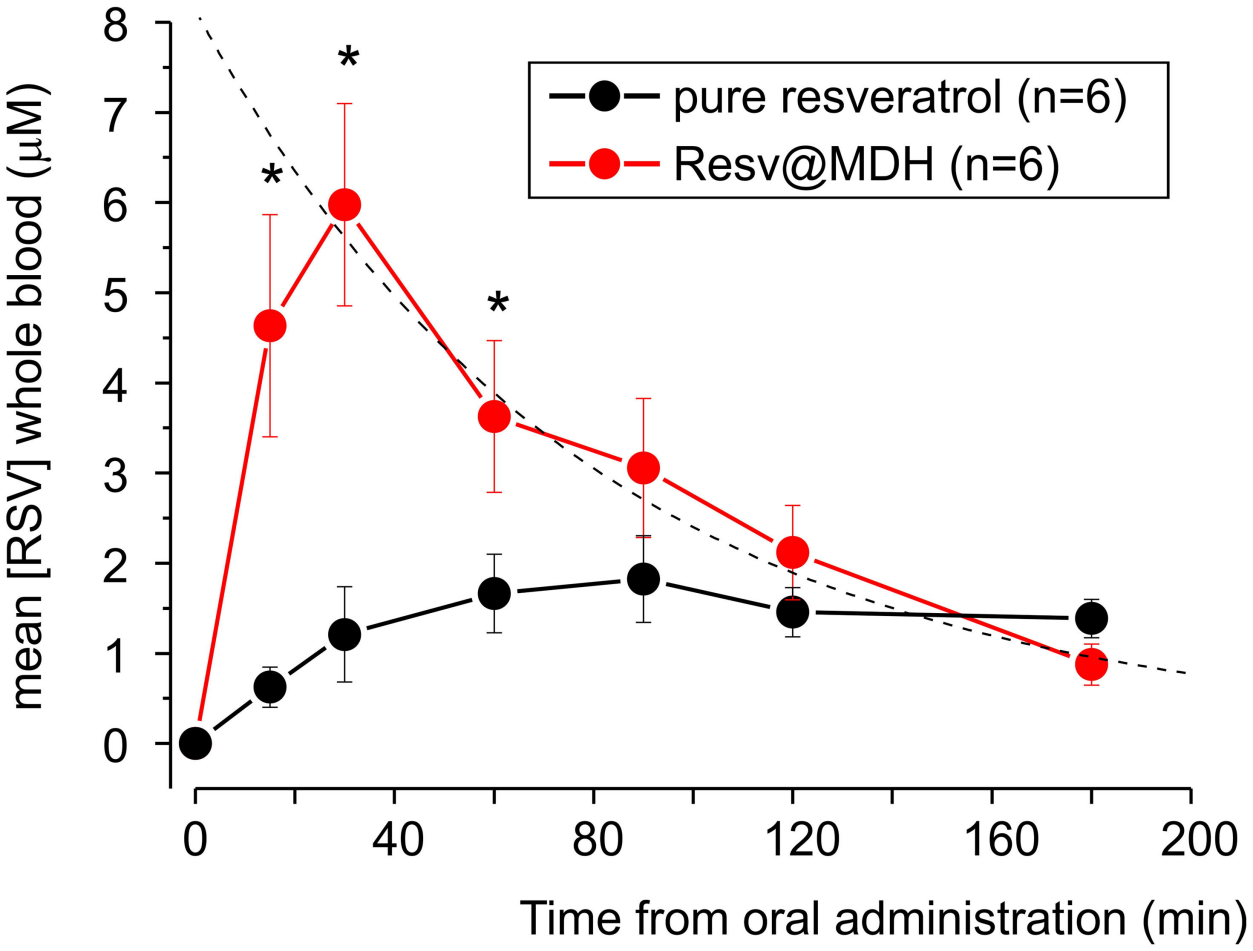
Mean plasma concentration-time curve for free plasmatic resveratrol (*n* = 6) after treatment with of either 180 mg pure resveratrol (Polygonum cuspidatum 98%) or 180 mg resveratrol from Resv@MDH obtained from subjects displayed in Figure 4. The dash line represents the best fit of the experimental data point of resveratrol blood concentration following Resv@MDH administration by a mono-exponential model (for details see text in Results section). The black and red dots indicate the resveratrol plasma concentration from the administration of pure resveratrol and Resv@MDH, respectively. *p < 0.05.

In Figure 5, we report the mean concentration of resveratrol in the whole blood as a function of time, combining the results from all subjects. By examining these data, we can specify the differences between the concentration profiles of pure resveratrol and Resv@MDH. A different pharmacokinetic profile was clear between the two sources of resveratrol. Concentration of pure resveratrol increased slowly after oral administration with a steady-state level at around 90 min, whereas with Resv@MDH, a transient profile was observed. Peak concentration occurred at around 30 min after oral administration, with a decay of plasma concentration best described by a mono-exponential model. The dash line in Figure 5 represent the best fit of experimental data point of resveratrol blood concentration following Resv@MDH administration with the equation [Resv]^*^exp((–t)/τ) + C with [Resv] maximal theoretical resveratrol concentration at *t* = 0, τ decay constant and C a constant that describes the resveratrol plasma concentration a infinite time. The parameter obtained is [Resv]= ca. 8 μM, τ = ca. 79 min and C = 130 nM.

The maximum plasma concentration (Cmax) of resveratrol was 2.2 and 6.3 μM for resveratrol and Resv@MDH, respectively (*p* = 0.01). For a time range spanning from 10 to 100 min after oral administration, plasma concentration of Resv@MDH-treated subjects was statistically greater than those treated with pure resveratrol (Figure 5). The Area Under Curve (AUC *t* = 0–3) values of the plasma concentration profile up until the 3-h mark were 248 ± 48 nmol/ml^*^min (*n* = 6) and 526 ± 92 nmol/ml^*^min (*n* = 6) for resveratrol and Resv@MDH, respectively. The data demonstrates an approximate 2-fold enhancement of resveratrol's bioavailability (*p* = 0.024) in the early phase of absorption (ratio of AUC t = 0–3 Resv@MDH/ AUC *t* = 0–3 resveratrol, Table 2). Interestingly, the AUC infinite similarly indicates that the differences between these formulations must be interpreted from a kinetic, not stationary, point of view (data not shown).

### Simulation of Pharmacokinetic Properties of Resv@MDH and Pure Resveratrol

**Figure 6:**
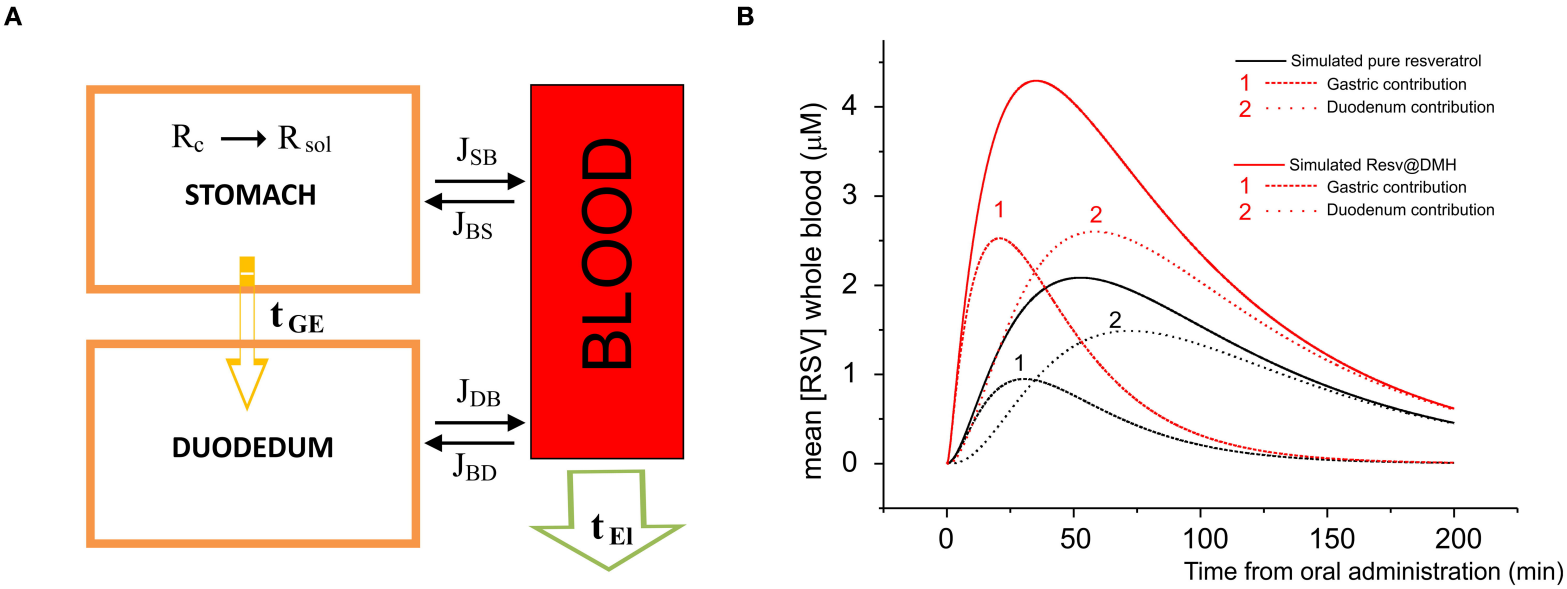
**(A)** Model of the absorption and elimination of resveratrol occurring in stomach, duodenum, and blood apparatus. Dissolution of resveratrol starts in the stomach and is indicated by R (s) → R (aq); resveratrol is transported toward the duodenum according to a gastric empty time τGE; the water solution of resveratrol is absorbed by the blood apparatus through stomach and duodenum membranes, according to the dissolution time and the gastric empty time; the passage (flux) from the stomach to the blood apparatus and vice versa, and from the duodenum to the blood apparatus and vice versa is indicated by J_SB_, J_BS_, J_DS_, J_SD_, respectively. Finally, resveratrol is eliminated or redistributed to other parts of the body, according to an elimination time τEl. **(B)** Concentration in μM as a function of the time of pure resveratrol (black line) and Resv@MDH (red line) in plasma. Dots indicate the experimental data, while continuous curves denote the data obtained by our model. For both pure resveratrol and Resv@MDH, the contribution to the plasma concentration due to gastric and duodenum absorption are reported. The number indicates the gastric (1) and duodenal (2) contributions on both simulations.

To underline the observed pharmacokinetic differences, we developed a simulation process where the unique differences are the dissolution properties described in Figure 2. In Figure 6A, we report a schematic model of the absorption and elimination/delivery of resveratrol in the human body. The absorption process starts with the dissolution of resveratrol in the stomach (see The dissolution rates of Resv@MDH and pure resveratrol section) and is, arguably, completed within the first segment of the intestine, the duodenum, where resveratrol is transported after a gastric empty time τ_GE_ (all the parameters employed in the model are reported in Table 3).

**Table 3:** The parameters employed in Equations (6)–(8).

**V_**STOMACH**_ (dm^**3**^)**	**V_**DUODENUM**_ (dm^**3**^)**	**V_**PLASMA**_ (dm^**3**^)**	**Dose coefficient**	**S_**S**_ (m^**2**^)**	**S_**D**_ (m^**2**^)**
0.25	3	5	3	0.02	0.2
**τ_GE_** **(s**^**−1**^**)**	**τ_EL_** **(s**^**−1**^**)**	**P**_**S**_ **(m·s**^**−1**^**)**	**P**_**D**_ **(m·s**^**−1**^**)**	**r**_**S**_	**r**_**D**_
50	79	2·10^−5^	1·10^−6^	3.15	3.15

*V_stomach_, The volume of the stomach; V_duodenum_, the volume of the duodenum; V_plasma_, the volume of plasma; S_S_ and S_D_, the dose, the surface of the stomach and duodenum, respectively, τ_GE_, the gastric empty time; τ_El_, the elimination time; P_S_, the permeability coefficient of stomach; P_D_ the permeability coefficient of the duodenum*.

Simultaneously, the water solution is diffused through the membranes of the stomach and duodenum toward the blood compartment. The corresponding flux in the unit time J(t), in mol s^−1^, is derived by the law of Fick:

(6) $$\begin{array}{l} \text{J}({\text{t}}_{\text{i}})\text{=}\sum {}_{\text{i=1}}^{\text{N}}\{{\text{P}}_{\text{S}}{\text{S}}_{\text{S}}{\text{r}}_{\text{S}}[\left({\text{mol}}_{\text{R}}({\text{t}}_{\text{i}})-\sum {}_{\text{l=1}}^{\text{i}-\text{1}}\text{J}({\text{t}}_{\text{l}})\right)\frac{\text{1}}{{\text{V}}_{\text{stomach}}}\\ -\sum {}_{\text{l=1}}^{\text{i}-\text{1}}\text{J}({\text{t}}_{\text{l}})\frac{\text{1}}{{\text{V}}_{\text{plasma}}}]{\text{e}}^{-\frac{{\text{t}}_{\text{i}}}{\tau_{\text{GE}}}}+{\text{P}}_{\text{D}}{\text{S}}_{\text{D}}{\text{r}}_{\text{D}}[\left({\text{mol}}_{{\text{R}}_{\text{i}}}-\sum {}_{\text{l=1}}^{\text{i}-\text{1}}\text{J}({\text{t}}_{\text{l}})\right)\\ \frac{\text{1}}{{\text{V}}_{\text{duodenum}}}-\sum {}_{\text{l=1}}^{\text{i}-\text{1}}\text{J}({\text{t}}_{\text{l}})\frac{\text{1}}{{\text{V}}_{\text{plasma}}}]\left(\text{1}-{\text{e}}^{-\frac{{\text{t}}_{\text{i}}}{\tau_{\text{GE}}}}\right)\} \end{array}$$

where P_S_ and P_D_ are the permeability coefficients in stomach and duodenum, respectively, and are: directly proportional to the diffusion and repartition coefficients and, inversely proportional to the thickness of the membrane, S_S_ and S_D_ are the surfaces of the stomach and duodenum wall, respectively; mol_R_(t_i_) is the number of mol of resveratrol (multiplied by the dose) in stomach at the time i, the summation over J(t_l_) is the total number of mol of resveratrol absorbed by the blood through the stomach and duodenum membranes; V_stomach_, V_duodenum_, and V_plasma_ are the volumes of stomach, duodenum and plasma, respectively. The terms in quadratic parenthesis indicate the gradient concentration of: stomach and plasma, and duodenum and plasma; the sign of the gradient indicates the sense of the flux. Finally, the exponential terms ${\text{e}}^{-\frac{{\text{t}}_{\text{i}}}{\tau_{\text{GE}}}}$ and 1– ${\text{e}}^{-\frac{{\text{t}}_{\text{i}}}{\tau_{\text{GE}}}}$ are weight functions which sum is 1 and represent the gastric empty and the duodenum filling, respectively; τ_GE_ indicates the gastric empty time.

The final stage consists in the elimination of resveratrol from the blood compartment, or in the redistribution toward other organs. The kinetics of this process follows an exponential decay

(7) $$\begin{array}{r} \left[\text{R}\right]=\left[{\text{R}}_{\text{max}}\right]{\text{e}}^{-\frac{{\text{t}}_{\text{i}}}{\tau_{\text{El}}}} \end{array}$$

where [R_max_] is the initial concentration of resveratrol and τ_El_ is the elimination time from the blood apparatus.

The concentration of resveratrol in plasma as a function of time is therefore given by the following equation:

(8) $$\begin{array}{r} {\left[\text{R}\right]}_{\text{plasma}}\left({\text{t}}_{\text{i}}\right)=\frac{1}{{\text{V}}_{\text{plasma}}}\text{J}\left({\text{t}}_{\text{i}}\right)\cdot {\text{e}}^{-\frac{{\text{t}}_{\text{i}}}{\tau_{\text{El}}}} \end{array}$$

In Figure 6B, we report the plasma concentration as a function of time. The plot compares the experimental data of pure resveratrol and Resv@MDH and those calculated by the related model from Equation (8). For each product, we have calculated the gastric and duodenum component. These findings regarding the pecular dissolution of resveratrol could explain the better gastric absorption of Resv@MDH.

## Discussion

Resveratrol is a natural molecule that is absorbed quickly along the digestive tract due to its optimal coefficient partition that allows it to maintain a balance between the water compartment and the biological membranes. Several studies have shown that about 80% of an orally administered dose of 25 mg is absorbed systemically and subsequently eliminated through urine (26). In contrast to this favorable absorption, limited bioavailability has been reported for resveratrol as it is rapidly metabolized to sulfates and glucoronidas (11, 26). Specifically, both pre-systemic metabolic activities (gut and liver) were reported through the *in vitro* study of the Caco-2 model (27), as well as systemic metabolism through the pharmacokinetic study of intravenous doses (11, 26).

Due to the metabolism, the plasma half-life of resveratrol following oral administration is about 2–4 h. Additional studies have shown that plasma concentration largely depends on the administered dose. In a dedicated study using single doses of 0.5, 1, 2.5, and 5 grams of resveratrol, maximum plasma concentrations (Cmax), varying from 73 to 539 ng/ml, were reported [0.3–2.4 μM, (28)]. At lower doses, a similar relationship between dose and plasma peak was also found.

After the first administration of 25, 50, 100, and 150 mg of resveratrol, Almeida et al. (18) observed a Cmax between 1.48 and 24.8 ng/ml with a peak time between 0.8 and 1.3 h. In agreement with Almeida et al. 400 and 500 mg of orally administered resveratrol reach a Cmax of 47 ng/ml (20) (200 nM) and 71 ng/ml (about 300 nM) (15), respectively. In Nunes et. (19), oral administration of 200 mg of resveratrol is comparable to that used in our study (180 mg) and the plasma concentration in elderly and young subjects of both sexes is around 25 ng/ml (about 100 nM), with a peak time ranging from 0.8 to 1.1 h and with a plasmatic half-life (*t*/2) of about 3 h. In our study, we found that the administration of 180 mg of pure resveratrol results in a blood peak concentration of about 2 μM with a latency of 115 min. The main difference between our study and the previous ones, mentioned above, is that we studied the total resveratrol concentration (plasmatic plus cellular) rather than just plasmatic. In fact, recent work on the rat animal model has shown that the amount of absorbed resveratrol is actually underestimated by about 76%, if the considered resveratrol concentration only accounts for that distributed in plasma and does not take into account the cellular component (21).

Although, there are no specific studies conducted in human subjects, we believe that the differences we observed are due to the different mode of blood sampling (whole blood vs. plasma). Dedicated experiments will be needed to discern the apparent differences between our data and those present in literature. Another factor that should be taken into account is the fasting state (our case), which increases the Cmax of resveratrol compared with the presence of food (20). Mode of administration is also important to consider, as done in our study, where both pure resveratrol and Resv@MDH were administered after dispersion of the powder in water.

We previously described the biophysical properties of resveratrol when supported by magnesium hydroxide (MDH). Specifically, we described microscopic properties by observing that, when resveratrol is dispersed into an array of magnesium hydroxide, it is composed of two types of microparticles: one of pure resveratrol and the other in the form of a complex with magnesium (22). Hydroxide magnesium is a safe matrix that dissolves rapidly in acidic settings such as the gastric one, through an acid-based reaction. An important result of this study is that resveratrol, when supported by the magnesium hydroxide matrix (Resv@MDH), shows a completely different plasma profile than that of pure resveratrol. When Resv@MDH is administered, a very early plasma peak (25 min) and a blood concentration of about 6 μM is present. Furthermore, there is greater bioavailability, measured through the AUC calculated on experimental points up to 180 min, by about twice the amount, when compared with that of pure resveratrol. This observation is similar to what has been previously observed in the animal model (22). It is interesting to note that the absolute bioavailability does not change, indicating that the differences are only of kinetic type. Early attainment of the plasma peak allows us to analyse and estimate the half-life time of resveratrol, which we report as being, on average, 80 min. This half-life component is most likely due to processes of elimination and distribution of resveratrol, and must be analyzed in detail in future works.

To understand whether the different pharmacokinetic behavior was due to the dissolution profile, we developed an absorption model where the only differences were in the dissolution profiles derived from the experiments, shown in Figure 2. The model consisted of three compartments: gastric, intestinal and blood. Gastro-enteric emptying was described by estimated mono exponential kinetics in a liquid meal emptying condition, while absorption from the stomach and intestine was described by the law of Fick.

The half-life of the total plasma resveratrol, obtained by the pharmacokinetic data of Figure 5, was mono-exponential based. We observe that the different properties of resveratrol based on the dissolution in the gastric environment explain the pharmacokinetic differences between pure resveratrol and Resv@MDH. Specifically, resveratrol with the dissolution properties of the Resv@MDH, in the gastric environment is expected to increase the plasma peak and decrease latency as observed experimentally.

Although the model is a simplification of the *in vivo* condition, it is clear that resveratrol resulting from the administration of Resv@MDH has a mainly gastric absorption. This is in contrast with resveratrol in its pure form, which is predominantly absorbed intestinally (see Figure 6). Another aspect we wish to emphasize is that, although the model shows that the main pharmacokinetic differences are due to the peculiar features in the process of dissolution in the gastric environment, other factors must be considered to explain such behaviors. When absorbed mostly at the gastric level, resveratrol could be subject to a different pre-systemic metabolism, compared to that of the model for the intestinal absorption (11). Further studies are required to assess these factors.

In conclusion, resveratrol's increased dissolution rate in the gastric environment, and the factors that lead to such an occurrence, should be leveraged to improve its absorption and to achieve a higher plasmatic peak with less latency time. This improved kinetics could represent the basis for the development of protracted formulations by, for instance, combining resveratrol with different dissolution rates to obtain a higher plasma concentration stability within a given therapeutic window. Considering the numerous health benefits associated with the consumption of resveratrol, our future studies will focus on evaluating possible biological activities of these formulations.

## Data Availability Statement

The raw data supporting the conclusions of this article will be made available by the authors, without undue reservation.

## Ethics Statement

The studies involving human participants were reviewed and approved by Regional ethics committee review board (project number 12477/18, approved on 18/01/2018 by CEAS Umbria, Perugia Italy). Registered in the ClinicalTrials.gov Protocol Registration System (identifier: NCT04258306). The patients/participants provided their written informed consent to participate in this study.

## Author Contributions

BF, RI, and FP: conceptualization. BF and FP: formal analysis. AF and AL: analytical analysis. RS: dissolution test. CC, FP, and BF: numerical simulation analysis. AT, RR, MD, and AL: clinical sampling. RI, AF, RS, MD, and BF: data curation. LM, AF, FR, RR, and AL: investigation. BF and RI: writing—original draft preparation. BF and LL: supervision. BF: project administration and funding acquisition. All authors contributed to the article and approved the submitted version.

## Conflict of Interest

RS and BF are co-inventors of the patent EPO n EP20130425091. RI and LL are employees of S&R Farmaceutici S.p.A., who hold the rights and license of REVIFAST^®^. The remaining authors declare that the research was conducted in the absence of any commercial or financial relationships that could be construed as a potential conflict of interest.

## Supplementary Material

Supplementary Figure 1 Logarithm of resveratrol concentration in whole blood as a function of the time of the data displayed in Figure 4.
