# Supplementary figures and images for: Resveratrol Supported on Magnesium DiHydroxide (Resv@MDH) Represents an Oral Formulation of Resveratrol With Better Gastric Absorption and Bioavailability Respect to Pure Resveratrol
Source: Front Nutr. 2020 Nov 13;7:570047. doi: 10.3389/fnut.2020.570047 (PMC8377765; doi:10.3389/fnut.2020.570047)

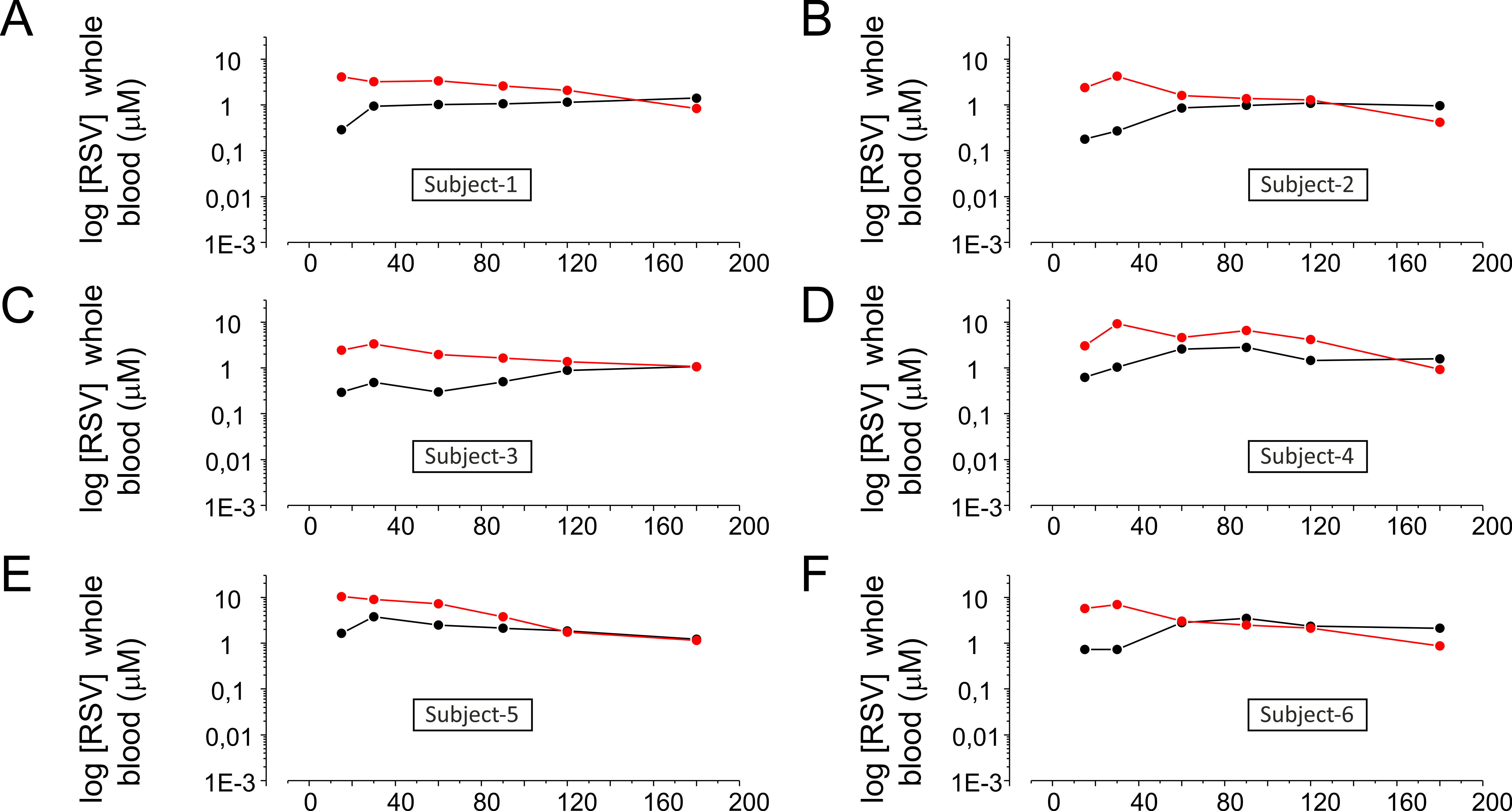

Supplement: Supplementary Figure 1 — Logarithm of resveratrol concentration in whole blood as a function of the time of the data displayed in Figure 4. [file Image_1.tif]
